# Circadian Influences on Chronic Kidney Disease Progression: Molecular Signaling Pathways of Melatonin and Their Therapeutic Potential

**DOI:** 10.3390/ph19060952

**Published:** 2026-06-18

**Authors:** Kuo-Cheng Lu, Chien-Lin Lu, Yi-Chou Hou, Yen-Sung Huang, Yu-Tien Chang, Cai-Mei Zheng, Chia-Chao Wu

**Affiliations:** 1 Division of Nephrology, Department of Medicine, Taipei Tzu Chi Hospital, Buddhist Tzu Chi Medical Foundation, New Taipei City 23142, Taiwan; kuochenglu@gmail.com; 2 School of Medicine, College of Medicine, Fu Jen Catholic University, New Taipei City 24205, Taiwan; 3 Division of Nephrology, Department of Internal Medicine, Fu Jen Catholic University Hospital, Fu Jen Catholic University, New Taipei City 24352, Taiwan; 4 Division of Nephrology, Department of Internal Medicine, Cardinal-Tien Hospital, New Taipei City 23155, Taiwan; 5 Institute of Biomedical Sciences, Academia Sinica, Taipei 115201, Taiwan; yshuang@ibms.sinica.edu.tw; 6 School of Public Health, National Defense Medical University, Taipei 114201, Taiwan; 7 Division of Nephrology, Department of Internal Medicine, Shuang Ho Hospital, Taipei Medical University, New Taipei City 23561, Taiwan; 8 Division of Nephrology, Department of Internal Medicine, Tri-Service General Hospital, National Defense Medical University, Taipei 11490, Taiwan

**Keywords:** chronic kidney disease, circadian rhythm, melatonin, BMAL1, Nrf2, renal fibrosis, TGF-β1, NLRP3 inflammasome, SIRT1, chrono-nephrology

## Abstract

Chronic kidney disease (CKD) remains a leading cause of premature mortality and global disease burden, yet the molecular mechanisms underlying its progression are still incompletely understood. Accumulating evidence highlights circadian disruption as an underappreciated driver of CKD that warrants systematic re-examination. The kidney harbors an autonomous circadian oscillator, principally regulated by the CLOCK:BMAL1 transcription factor complex, which coordinates glomerular filtration, tubular electrolyte handling, blood pressure rhythmicity, inflammatory tone, and cellular repair. In CKD, retained uremic toxins, sustained oxidative stress, and persistent NF-κB activation collectively suppress this clock machinery, generating a self-reinforcing cycle of renal injury and circadian dysregulation. CKD is also accompanied by progressive attenuation of nocturnal melatonin secretion, weakening a central hormonal cue for peripheral clock entrainment and cytoprotection. Melatonin acts both as a chronobiotic and as a pleiotropic cytoprotective molecule. Through MT1/MT2 receptors, the nuclear receptor RORα, and receptor-independent antioxidant pathways, it may enhance Nrf2/HO-1 signaling, restrain NF-κB and NLRP3 inflammasome activity, suppress TGF-β1/Smad2/3-mediated fibrogenesis, preserve mitochondrial integrity, and engage SIRT1-linked clock regulation. Current clinical studies suggest that nightly melatonin supplementation can improve sleep quality and selected oxidative or circadian surrogate endpoints in hemodialysis patients; however, whether melatonin slows CKD progression or preserves renal function remains unproven. This review synthesizes the molecular interface between circadian dysregulation and CKD progression and articulates a rationale for adequately powered clinical trials evaluating melatonin as a candidate chronotherapeutic adjunct rather than an established renoprotective therapy.

## 1. Introduction

Chronic kidney disease (CKD) represents one of the most pressing global health challenges of the present era. Current epidemiological estimates indicate that more than 10% of the world’s population—approximately 700–843 million individuals—are affected, and CKD is now firmly established as an independent contributor to cardiovascular disease, cognitive deterioration, functional decline, and premature mortality [1]. Hypertension and diabetes mellitus remain the dominant global drivers of CKD, placing its prevalence and public-health burden in the same clinical ecosystem as these highly prevalent, socially significant diseases. Because hypertension, diabetes, obesity, sleep disturbance, and psychosocial stress are multifactorial and bidirectionally linked to circadian misalignment, stress-related disruption of sleep–wake, neuroendocrine, autonomic, and inflammatory rhythms may also contribute to the initiation or acceleration of CKD risk in susceptible individuals [1]. Despite decades of therapeutic progress centered on renin–angiotensin–aldosterone system (RAAS) blockade, glycemic control, blood pressure management, and dietary phosphate restriction, many patients continue to experience progressive nephron loss [2]. This persistent therapeutic gap reflects an incomplete mechanistic understanding of the molecular events that drive disease progression beyond proteinuria, hypertension, and diabetes alone.

Circadian biology offers a largely untapped mechanistic perspective from which to reinterpret CKD pathophysiology. The mammalian circadian clock is a cell-autonomous molecular oscillator operative in virtually all tissues, including the kidney, where it governs an estimated 10–20% of the renal transcriptome in a time-of-day-dependent manner [3]. The core clock proteins—CLOCK, BMAL1, PER1–3, and CRY1/2—constitute a transcription–translation feedback loop (TTFL) that generates approximately 24 h oscillations, and their downstream clock-controlled genes (CCGs) encode pivotal renal effectors, including the sodium–hydrogen exchanger NHE3, the sodium–chloride cotransporter NCC, and multiple components of the RAAS [4]. A critical translational insight from recent studies is that disruption of these rhythms is not a downstream consequence of CKD but an active driver of progression: collecting-duct-specific deletion of Bmal1 in the Pkd1RC/RC mouse model of autosomal dominant polycystic kidney disease (ADPKD) accelerates cyst growth, increases renal fibrosis, and disturbs lipid metabolism [5]. Patients with CKD exhibit progressive circadian transcriptomic dysregulation, with altered clock gene expression reported in CKD-related tissues and peripheral immune-cell transcriptomic profiles, suggesting that circadian disruption may worsen with disease progression [6].

Melatonin—the pineal indoleamine secreted at night in response to suprachiasmatic nucleus (SCN)-driven sympathetic signaling—functions concurrently as the principal endocrine zeitgeber for circadian alignment and as a direct cytoprotective molecule. Impaired melatonin secretion has been reported in patients with CKD and is associated with renal damage, while altered urinary 6-sulfatoxymelatonin (aMT6s) excretion has also been described in advanced renal dysfunction and dialysis populations [7,8]. This deficiency arises from uremic suppression of pineal AANAT activity, impaired renal clearance of melatonin metabolites, and disrupted light–dark entrainment within hospital and dialysis-unit environments [9]. The combined circadian–cytoprotective deficit establishes a pathological amplification loop in which clock disruption exacerbates renal oxidative stress and inflammation, which in turn further suppresses melatonin biosynthesis and clock gene expression.

Previous reviews have considered circadian biology in nephrology [10] or the antioxidant properties of melatonin [11] as separate domains. The present review integrates both within a unified molecular signaling framework, with three principal mechanistic emphases: (i) the molecular cascades through which circadian disruption accelerates CKD progression; (ii) the multi-target pharmacology of melatonin across the dominant pathomechanisms of CKD; and (iii) the emerging clinical evidence supporting melatonin as a chronotherapeutic agent, accompanied by a critical appraisal of the knowledge gaps that must be addressed before adequately powered trials can be designed.

## 2. Central Pacemaker Control, Molecular Clockwork, and Systemic Physiological Rhythms

The vertebrate circadian system is governed by a master pacemaker located in the suprachiasmatic nucleus (SCN) of the hypothalamus, which synchronizes peripheral clocks distributed throughout the body through neural, endocrine, behavioral, and temperature-related signals [12,13]. Light entrains the circadian system through retinal input to the SCN, after which timing information is transmitted to peripheral organs via autonomic output, hormonal rhythms, sleep–wake behavior, feeding–fasting cycles, and body temperature oscillations. Molecular circadian timekeeping is maintained by transcription–translation feedback loops involving CLOCK, BMAL1, PER, and CRY, thereby regulating sleep–wake behavior, metabolism, and hormonal rhythms [14]. Circadian regulation extends across cardiovascular, metabolic, endocrine, renal, and immune systems, including rhythms in blood pressure, glucose handling, glucocorticoid secretion, renal function, cytokine activity, and immune surveillance [14,15].

### 2.1. Molecular Architecture of the TTFL

The mammalian circadian clock is built upon interlocking transcription–translation feedback loops (TTFLs). In the primary loop, the heterodimeric transcription factor CLOCK:BMAL1 binds E-box enhancer elements and initiates rhythmic transcription of Per1/2/3 and Cry1/2. PER and CRY proteins then accumulate in the cytoplasm, form inhibitory complexes, and translocate back into the nucleus, where they suppress CLOCK:BMAL1-driven transcription. This delayed negative feedback generates an approximately 24 h oscillation. Temporal precision is refined by post-translational regulation: casein kinase 1δ/ε (CK1δ/ε) phosphorylates PER proteins and targets them for β-TrCP-dependent proteasomal degradation, whereas FBXL3 regulates CRY stability [16]. A secondary stabilizing loop is formed by REV-ERBα/β and RORα/γ, which compete at ROR-response elements in the Bmal1 promoter and create antiphasic Bmal1 oscillation. For nephrologists, the key translational point is that this clock is not only a timing device; it imposes daily rhythmicity on transporter expression, mitochondrial metabolism, inflammatory readiness, blood pressure regulation, and repair programs. SIRT1-mediated deacetylation of BMAL1 and PER2 further connects NAD^+^ availability, cellular energy status, and circadian amplitude, providing a mechanistic bridge to the melatonin-related pathways discussed below.

Conceptually, CLOCK:BMAL1 functions as the “accelerator” phase of the clock, PER/CRY complexes provide the delayed “brake,” and REV-ERB/ROR transcription factors stabilize the next cycle of Bmal1 expression. Disruption at any of these levels can flatten rhythmic gene expression and convert normally time-gated renal responses into persistent injury signals, particularly under uremic, oxidative, or inflammatory stress.

### 2.2. Clock Gene Expression and Function in the Kidney

The kidney possesses intrinsic peripheral circadian clocks that regulate tubular transport, metabolic activity, electrolyte balance, and blood pressure control [17,18]. Experimental and omics-based studies indicate that renal circadian regulation is segment-specific, with clock-dependent activity observed in the proximal tubule, distal nephron, and collecting duct [17,19]. In proximal tubular cells, BMAL1-dependent circadian signaling may modulate organic anion transport pathways, including OAT1/OAT3-mediated handling of drugs and uremic solutes, thereby linking the renal clock to tubular secretory function [19]. In parallel, clock-regulated sodium transporters, including NHE3, NCC, NKCC2, and ENaC, provide a mechanistic link between circadian renal sodium handling and daily blood pressure rhythmicity [20,21,22]. These pathways are introduced here as normal physiological clock-output systems; their disruption in CKD is discussed in the following sections.

### 2.3. Circadian Regulation of Renal Hemodynamics, RAAS Oscillation, and Immune–Oxidative Homeostasis in CKD

In healthy humans, renal plasma flow, glomerular filtration rate, and tubular transport exhibit marked circadian variation, with values fluctuating by approximately 20–40%, peaking in the early afternoon and reaching their nadir during nocturnal sleep. These renal rhythms are partly coordinated through circadian regulation of the RAAS, as plasma renin activity, angiotensin II, and aldosterone peak between 03:00 and 06:00, coinciding with the physiological morning blood pressure surge. This RAAS oscillation is dependent on clock gene expression in juxtaglomerular and adrenal fasciculata cells [23]. In CKD, circadian disruption, potential uremic-toxin-related chronodisruption, altered intrarenal RAAS rhythmicity, and increased sympathetic nerve activity may contribute to elevated nocturnal blood pressure and a non-dipping phenotype [24,25,26]. This non-dipping phenotype is clinically important because reduced nocturnal BP decline and elevated nocturnal BP are associated with cardiovascular risk and CKD progression, partly independent of average ambulatory or 24 h BP burden [26,27].

**Figure 1:**
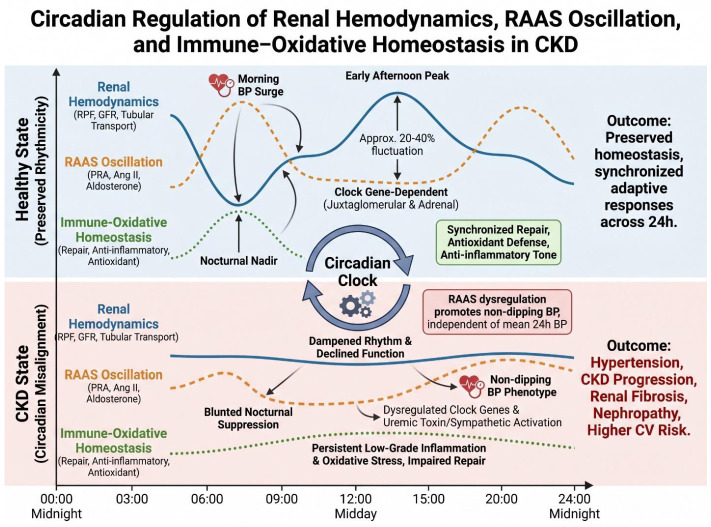
Circadian clock architecture and renal chronobiology in CKD. This figure summarizes the hierarchical organization of the vertebrate circadian system and its relevance to renal physiology and CKD. Light input from the retina entrains the suprachiasmatic nucleus (SCN), which coordinates peripheral clocks through endocrine and autonomic signaling. At the molecular level, CLOCK:BMAL1 activates Per and Cry transcription through E-box elements; PER/CRY complexes subsequently repress CLOCK:BMAL1 activity, forming the core transcription–translation feedback loop. Additional regulatory layers include CK1δ/ε-mediated PER degradation, FBXL3-dependent CRY stability, REV-ERB/ROR control of Bmal1, and SIRT1-mediated metabolic–clock coupling. In the kidney, intrinsic circadian clocks regulate tubular transport, electrolyte handling, renal hemodynamics, RAAS oscillation, immune–oxidative homeostasis, and blood pressure rhythmicity. Circadian disruption in CKD may impair these coordinated rhythms, contributing to nocturnal hypertension, non-dipping blood pressure, inflammation, oxidative stress, fibrosis, cardiovascular risk, and CKD progression.

Beyond hemodynamic control, the kidney possesses an intrinsic circadian clock that regulates tubular transport, electrolyte handling, immune–inflammatory tone, antioxidant defense, mitochondrial homeostasis, and repair responses. Disruption of this rhythmic coordination is associated with hypertension, CKD progression, renal fibrosis, and nephropathy [3]. Circadian misalignment may impair kidney immunity by disturbing leukocyte trafficking, cytokine signaling, and cellular repair timing, thereby promoting persistent low-grade inflammation and amplifying oxidative stress, both of which contribute to renal injury progression and cardiovascular complications in CKD [3,28]. Conversely, preserved circadian rhythmicity may support renal homeostasis by synchronizing anti-inflammatory, antioxidant, hemodynamic, and tubular adaptive responses across the 24 h cycle [3,29] (Figure 1).

## 3. Melatonin: Biosynthesis, Receptors, and Core Signaling Mechanisms

### 3.1. Biosynthetic Pathway and Extrapineal Production

Melatonin biosynthesis proceeds through four sequential enzymatic steps: hydroxylation of tryptophan by TPH1/2 yields 5-hydroxytryptophan; decarboxylation by AADC produces serotonin; N-acetylation by AANAT generates N-acetylserotonin; and finally O-methylation by ASMT/HIOMT produces melatonin. The rate-limiting AANAT step is acutely activated at night through sympathetic signaling: β2-adrenergic receptor engagement elevates cAMP, activates protein kinase A (PKA), and stabilizes the AANAT protein, while AANAT activity is suppressed during daylight. Importantly, extrapineal melatonin synthesis occurs in renal tubular cells, gastrointestinal enterochromaffin cells, and immune cells, generating local tissue concentrations that may exceed systemic plasma levels by an order of magnitude or more—an observation with significant implications for paracrine renoprotection independent of the pineal axis [30].

### 3.2. Receptor Subtypes and Downstream Coupling

Melatonin produces its biological effects through three major mechanisms: activation of membrane G-protein-coupled receptors, modulation of nuclear receptor signaling, and direct receptor-independent antioxidant activity. Its main membrane receptors, MT1 and MT2, are high-affinity GPCRs that regulate intracellular signaling pathways, including inhibition of cAMP-dependent signaling, and contribute to circadian regulation and other tissue-specific physiological effects [31,32,33]. In addition to membrane receptor signaling, melatonin may influence nuclear-receptor-related pathways, including RORα-associated transcriptional regulation, which has been implicated in inflammation and immune responses [34,35]. Melatonin also acts independently of receptors as a potent antioxidant. Through direct free-radical scavenging, it neutralizes reactive species such as hydroxyl radicals and peroxynitrite and generates antioxidant metabolites including AFMK and AMK. This receptor-independent activity is particularly relevant in mitochondria, where melatonin can accumulate at concentrations higher than those found in plasma [31,36] (Table 1).

## 4. Circadian Disruption in CKD: Mechanisms and Evidence

### 4.1. Clinical and Epidemiological Evidence

Circadian disruption manifests clinically in CKD as sleep disturbance, restless legs syndrome, blunted core body temperature rhythms, and a blood pressure non-dipping phenotype; sleep abnormalities affect more than 60% of ESRD patients in polysomnographic studies. A 2024 longitudinal analysis reported that “circadian syndrome”—a clustered phenotype comprising abdominal obesity, dyslipidemia, hypertension, hyperglycemia, depression, and short sleep—was independently associated with incident CKD during four years of follow-up, with risk increasing as additional components accumulated [46]. This observation does not prove that circadian disruption alone causes CKD, but it supports the concept that metabolic disease, psychosocial stress, sleep disruption, and circadian misalignment may converge on kidney risk through shared inflammatory, vascular, and neuroendocrine pathways [6,46,47].

### 4.2. Uremic-Toxin-Mediated Clock Disruption

**Figure 2:**
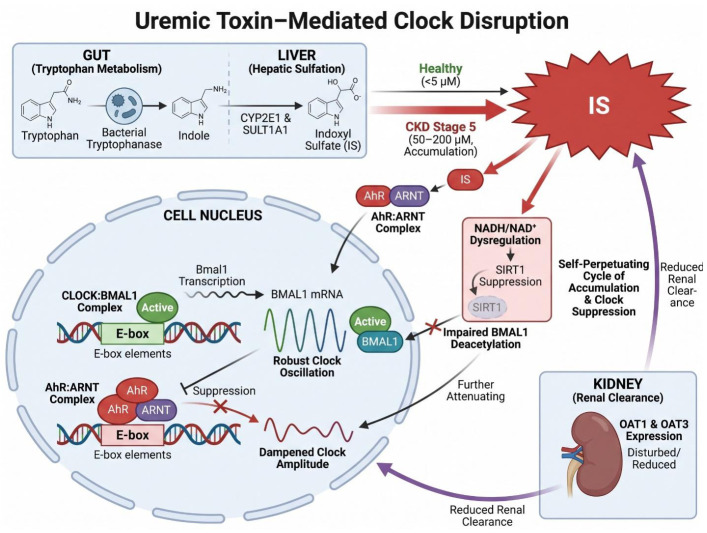
Clinical and molecular pathways linking circadian disruption to CKD progression. This figure illustrates the clinical and mechanistic pathways through which circadian disruption may contribute to CKD onset and progression. Clinically, CKD-related chronodisruption manifests as sleep disturbance, restless legs syndrome, blunted core body temperature rhythms, and a non-dipping blood pressure phenotype, which has been associated with renal and cardiovascular risk. Epidemiologically, circadian syndrome—defined by metabolic, cardiovascular, mood, and sleep-related abnormalities—has been linked to incident CKD, with risk increasing as more syndrome components accumulate. Mechanistically, gut-derived indoxyl sulfate accumulates in advanced CKD and may activate AhR-dependent signaling, suppressing CLOCK:BMAL1-mediated circadian transcription and reducing clock amplitude. Additional disruption may occur through SIRT1 suppression and altered NADH/NAD^+^ balance, further impairing BMAL1 regulation. OAT1/OAT3-mediated tubular secretion normally contributes to indoxyl sulfate handling, but reduced transporter function may promote toxin accumulation and worsen CKD-associated chronodisruption.

A central mechanism through which CKD perturbs circadian rhythms involves activation of the aryl hydrocarbon receptor (AhR) by IS and related indolic uremic toxins. IS is generated through tryptophan metabolism, beginning with bacterial conversion of tryptophan to indole by intestinal tryptophanase, followed by hepatic sulfation via CYP2E1 and SULT1A1; it is a high-affinity AhR ligand that accumulates to 50–200 μM in stage 5 CKD compared with less than 5 μM in healthy individuals [48]. Once activated, AhR:ARNT complexes compete with CLOCK:BMAL1 for shared E-box elements, directly suppressing Bmal1 transcription and dampening clock amplitude. This effect is compounded by IS-mediated suppression of SIRT1 through dysregulation of the NADH/NAD^+^ ratio, which impairs BMAL1 deacetylation and further attenuates clock oscillation [49] (Figure 2).

OAT1 and OAT3 play a central role in the renal handling of uremic toxins and solutes, including gut-microbiome-derived metabolites such as indoxyl sulfate [50]. Reduced transporter expression or activity may impair tubular secretion of organic anions and thereby promote toxin accumulation in CKD. However, direct evidence that indoxyl sulfate itself disrupts the circadian expression of OAT1/OAT3 remains insufficient. Accordingly, the OAT1/OAT3–indoxyl sulfate–clock axis should be regarded as a testable hypothesis: reduced transporter function may increase uremic toxin exposure, while toxin-induced AhR and oxidative signaling may further dampen CLOCK:BMAL1 activity and indirectly impair tubular secretory programs [50,51].

### 4.3. BMAL1 as a Causal Driver: Genetic Evidence

The strongest experimental evidence for clock disruption as a causal contributor to kidney disease progression comes from genetic models. A 2025 study in the *Journal of the American Society of Nephrology* demonstrated that collecting-duct-specific Bmal1 deletion in the Pkd1RC/RC mouse model of ADPKD accelerated cyst growth, increased renal fibrosis scores, and dysregulated lipid metabolism in a manner consistent with disturbed cellular energy homeostasis [5]. BMAL1 has also been implicated across the AKI-to-CKD transition, particularly in maladaptive tubular repair processes such as partial epithelial-to-mesenchymal transition and G2/M cell-cycle arrest [49]. G2/M arrest is considered fibrogenic because incompletely repaired tubular epithelial cells can remain metabolically active but fail to complete regeneration; instead, they secrete profibrotic mediators, including TGF-β1 and connective tissue growth factor (CTGF), which recruit fibroblasts, promote myofibroblast activation, and enhance extracellular matrix deposition. Thus, clock disruption may influence fibrosis not only through altered transport or hemodynamics, but also by changing the timing and quality of tubular repair.

### 4.4. Melatonin Deficiency as a Consequence and Amplifier of Circadian Disruption

**Figure 3:**
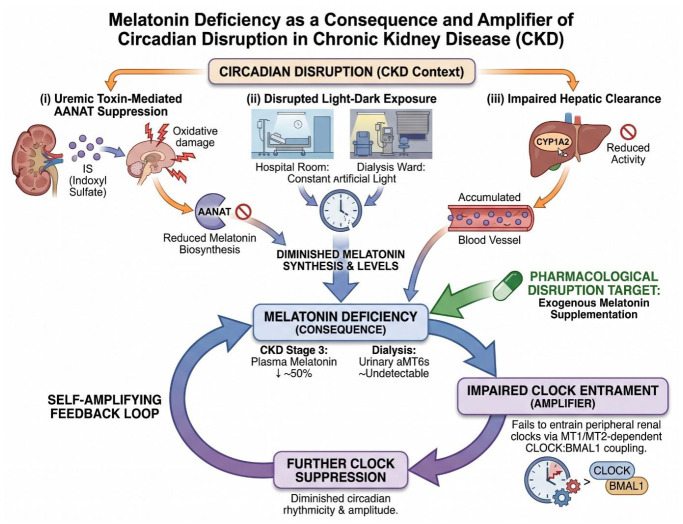
Melatonin deficiency as a consequence and amplifier of circadian disruption in CKD. This figure illustrates how CKD-associated chronodisruption and melatonin deficiency interact in a self-amplifying cycle. In CKD, nocturnal melatonin secretion progressively declines, with marked reductions evident by stage 3 and near absence of urinary aMT6s excretion in dialysis-dependent patients. Contributing mechanisms include uremic-toxin-mediated suppression of pineal melatonin synthesis, disrupted environmental light–dark cues, and impaired melatonin metabolism in advanced kidney disease. Reduced melatonin availability weakens MT1/MT2-mediated entrainment of peripheral renal clocks, thereby aggravating CLOCK:BMAL1 dysregulation and further impairing circadian homeostasis. This vicious cycle may promote CKD progression, and exogenous melatonin supplementation is identified as a potential therapeutic strategy to restore circadian alignment and renal protection.

Clinical studies report that nocturnal plasma melatonin concentrations are reduced by approximately 50% by CKD stage 3, while urinary 6-sulfatoxymelatonin (aMT6s), the major excreted melatonin metabolite, becomes nearly undetectable in many dialysis-dependent patients [9]. These values should be interpreted as study-specific estimates because absolute melatonin and aMT6s concentrations vary with assay platform, sampling time, light exposure, age, and dialysis schedule. Several mechanisms may contribute: (i) uremic-toxin-mediated suppression of pineal AANAT, the rate-limiting enzyme of melatonin biosynthesis, through oxidative injury to pinealocytes; (ii) disrupted light–dark exposure within hospital and dialysis-ward environments; and (iii) altered hepatic metabolism in advanced CKD. Melatonin is primarily metabolized by hepatic CYP1A2 to 6-hydroxymelatonin, which is subsequently conjugated and excreted as aMT6s; therefore, reduced CYP1A2 activity and impaired renal elimination of metabolites may prolong or distort melatonin pharmacokinetics while still leaving patients with an attenuated endogenous nocturnal rhythm [8,9]. Because melatonin can entrain peripheral clocks through MT1/MT2-dependent signaling, deficiency of the nocturnal melatonin signal may weaken renal clock alignment and contribute to a self-amplifying loop of chronodisruption, oxidative stress, inflammation, and impaired tubular repair (Figure 3).

## 5. Molecular Signaling Pathways of Melatonin in CKD Pathomechanisms

This section examines, in mechanistic detail, how melatonin engages the principal molecular cascades that drive CKD progression. Each subsection follows a uniform structure: (a) the pathway’s significance in CKD; (b) melatonin’s primary mechanistic interventions; and (c) supporting experimental evidence.

### 5.1. Nrf2/Keap1/ARE Axis: Antioxidant Defense

Uremic oxidative stress in CKD arises through several converging mechanisms, including IS-mediated activation of NADPH oxidase (NOX2/NOX4), uncoupling of mitochondrial electron transport at Complexes I and III, and progressive depletion of reduced glutathione (GSH), superoxide dismutase (SOD), and catalase. Nuclear factor erythroid 2-related factor 2 (Nrf2)—the master transcriptional regulator of antioxidant gene expression—is paradoxically suppressed in CKD despite elevated reactive oxygen species (ROS), primarily as a result of Keap1-independent Nrf2 degradation mediated by AhR-driven CUL3/Rbx1 ubiquitin ligase activity and IS-induced hypermethylation of the Nrf2 promoter [52,53,54].

Melatonin may enhance Nrf2/HO-1 antioxidant signaling through several complementary mechanisms, but direct biochemical evidence that melatonin itself modifies specific Keap1 cysteine residues in renal cells is currently insufficient. Accordingly, melatonin is best described as potentially promoting Nrf2 signaling through receptor-independent antioxidant effects, MT1/MT2-associated Akt/GSK-3β regulation, and SIRT1-related deacetylation mechanisms that favor Nrf2 nuclear retention and ARE-dependent transcription. Genes potentially upregulated through this melatonin–Nrf2 axis include HO-1, NQO1, GCLC, GPx1, PRDX3, and thioredoxin reductase 1 [52,55]. In a 2024 diclofenac-induced acute kidney injury rat model, melatonin exerted nephroprotective effects by suppressing renal miR-34a expression, enhancing Nrf2/HO-1/GSH antioxidant signaling, and attenuating NLRP3 inflammasome activation [56]. A related non-renal spinal cord injury study also supports the broader concept that melatonin can inhibit NLRP3 inflammasome activation through stimulation of the Nrf2/ARE pathway, but this evidence should be interpreted as cross-organ rather than direct CKD evidence [57].

### 5.2. NF-κB Pathway: Suppression of Renal Inflammation

Nuclear factor kappa-light-chain-enhancer of activated B cells (NF-κB) is a central transcriptional coordinator of the sterile inflammatory response in CKD. IS engages TLR4 and RAGE on proximal tubular cells and macrophages, triggering IKKα/β-mediated phosphorylation and ubiquitin-dependent degradation of IκBα, followed by nuclear translocation of the p65/p50 heterodimer and transcription of IL-6, IL-1β, TNF-α, MCP-1, iNOS, and COX-2. Critically, NF-κB p65 binds directly to the Bmal1 promoter and represses its transcription, providing a mechanistic bridge that links the inflammatory cascade to circadian clock disruption [58].

Melatonin restrains NF-κB activity through several complementary and context-dependent routes. MT1- and MT2-mediated Gαi/o coupling can reduce cAMP-dependent signaling, while melatonin-driven antioxidant effects may indirectly limit IKK activation by decreasing ROS-sensitive inflammatory signaling. In some experimental systems, melatonin has also been reported to reduce p65 nuclear activity and DNA-binding capacity. In addition, Nrf2-driven HO-1 upregulation may generate carbon monoxide and other cytoprotective signals that negatively regulate IKKβ. Thus, the Nrf2–NF-κB counter-regulatory axis provides a plausible anti-inflammatory framework for melatonin in CKD, although the relative contributions of receptor-dependent, antioxidant, and cell-type-specific mechanisms remain to be defined [57,59,60,61].

### 5.3. NLRP3 Inflammasome: Pyroptosis and IL-1β/IL-18 Suppression

The NLRP3 inflammasome—comprising the sensor NLRP3, the adaptor ASC, and the effector caspase-1—is increasingly recognized as a pivotal amplifier of CKD progression [62]. NLRP3 activation in renal tubular cells and macrophages requires two sequential signals: a priming signal mediated by NF-κB-dependent transcriptional upregulation of NLRP3 and pro-IL-1β, and an activation signal driven by ROS, K^+^ efflux, or lysosomal rupture from crystalline deposits. Once assembled, caspase-1 cleaves pro-IL-1β and pro-IL-18 into their bioactive secreted forms and processes gasdermin D (GSDMD) into the pore-forming N-terminal fragment (GSDMD-N) that mediates pyroptotic cell death. In CKD, IS serves as a primary stimulus for NLRP3 activation by promoting mitochondrial ROS generation, thereby establishing a feed-forward inflammatory loop in which NOX4-derived ROS sustains NLRP3 activation, IL-1β release, and consequent NF-κB activation [63].

Melatonin may inhibit both NLRP3 priming—through suppression of NF-κB-dependent transcription of NLRP3 and pro-IL-1β—and NLRP3 activation by reducing mitochondrial ROS, preserving mitochondrial integrity, and strengthening Nrf2-linked antioxidant defenses. Evidence from renal and non-renal injury models supports an anti-inflammasome effect of melatonin, but renal tubular KCNQ1-specific regulation of IL-1β/IL-18 secretion by melatonin has not been directly established in CKD. The NLRP3-related conclusions are therefore framed as a supported but context-dependent anti-inflammatory effect of melatonin rather than as a proven ion-channel-specific pathway in CKD [57,59,60,61,62,63].

### 5.4. TGF-β1/Smad2/3: Anti-Fibrotic Mechanisms

Renal fibrosis is the final common pathway of progressive CKD, characterized by tubulointerstitial fibrosis, myofibroblast accumulation, α-SMA expression, and excessive deposition of collagen I/III/IV and fibronectin. TGF-β1 is the central fibrogenic cytokine: in CKD, IS-driven STAT3 phosphorylation increases TGF-β1 expression in injured tubular cells, activating the TβRI/II receptor complex, Smad2/3 phosphorylation, Smad4 assembly, nuclear translocation, and downstream pro-fibrotic gene transcription. In parallel, TGF-β1 activates non-Smad pathways, including TAK1–JNK/p38 and PI3K/Akt, which further promote extracellular matrix deposition and impair tubular epithelial regenerative capacity [64,65].

Melatonin counteracts profibrotic signaling at multiple levels [66,67]. Through receptor-independent ROS scavenging, melatonin may suppress oxidative amplification of STAT3- and TGF-β1-related signaling; through SIRT1-mediated Smad3 deacetylation, it may reduce Smad3 transcriptional activity; and through receptor-linked signaling, it may modulate kinase pathways that influence Smad phosphorylation [68]. Melatonin also activates SIRT1–FOXO3a signaling to upregulate Smad7, an endogenous inhibitor of TGF-β signaling, while combined melatonin and poricoic acid A treatment suppressed Smad3- and β-catenin-driven fibrogenesis in a 5/6 nephrectomy AKI-to-CKD model [69]. The prior membranous nephropathy study on PITX1-mediated transcriptional regulation of MTNR1A and its association with E-cadherin preservation and α-SMA suppression provides disease-context-specific evidence from a glomerular proteinuric model, not definitive proof of a universal tubular anti-fibrotic mechanism. Thus, this finding should be interpreted as supportive preliminary evidence that melatonin receptor signaling may intersect with epithelial-injury and profibrotic pathways, while broader conclusions about tubulointerstitial fibrosis require validation in dedicated tubular and CKD models [70,71,72].

### 5.5. PI3K/Akt/mTOR: Tubular Survival and Autophagy

The PI3K/Akt/mTOR evidence base is heterogeneous and requires cell-type- and disease-context-specific interpretation. Indoxyl sulfate has been shown to suppress Akt phosphorylation and activate AhR-dependent inflammatory signaling in endothelial and vascular smooth muscle contexts, but these findings should not be directly equated with proximal tubular biology without further validation [73,74]. In diabetic nephropathy, excessive mTORC1 activation contributes to podocyte injury and impaired autophagic homeostasis, whereas non-diabetic CKD and uremic tubular injury may involve overlapping but not identical mechanisms [75,76]. Melatonin may restore autophagy through receptor- and energy-sensing pathways, including MT1/AMPK activation, AMPK–mTOR–ULK1 signaling, and Beclin-1-associated autophagosome formation [77]. Melatonin also promotes mitochondrial quality control in diabetic nephropathy by enhancing AMPK phosphorylation and facilitating PINK1/Parkin-mediated mitophagy [78]. These studies support a plausible cytoprotective role for melatonin in renal injury, but diabetic podocyte or vascular findings should not be extrapolated as direct proof of benefit in non-diabetic uremic tubular CKD [79,80].

### 5.6. SIRT1/PGC-1α/AMPK: Mitochondrial Biogenesis and Metabolic–Clock Coupling

In CKD, impaired renal NAD^+^ biosynthesis may reduce the availability of NAD^+^ for SIRT1-dependent deacetylation, while PARP-1 overactivation may further consume intracellular NAD^+^ and functionally constrain SIRT1 activity [81,82]. Melatonin may restore mitochondrial homeostasis through MT1/SIRT1/PGC-1α- and AMPK/PGC-1α-dependent signaling, promoting mitochondrial biogenesis and improving mitochondrial function, including increased TFAM expression and enhanced respiratory-chain activity in experimental injury models [83,84]. Mechanistically, AMPK and SIRT1 act as complementary energy sensors that regulate PGC-1α through phosphorylation and deacetylation, respectively, thereby linking cellular energy stress to mitochondrial transcriptional programs [85]. This pathway may also connect mitochondrial energetics with circadian regulation because SIRT1 interacts with the CLOCK–BMAL1 complex, modulates core clock components, and participates in NAD^+^-dependent circadian control of mitochondrial oxidative metabolism [86,87,88]. The clinical relevance of this axis is supported by evidence that serum from hemodialysis patients can suppress endothelial SIRT1 protein expression and by clinical studies showing that melatonin administered around intradialytic exercise improves immune, oxidative, antioxidant, and inflammatory profiles in hemodialysis patients [89,90,91].

### 5.7. CLOCK:BMAL1 Restoration: Melatonin as a Renal Chronobiotic

Beyond its direct antioxidant and cytoprotective actions, melatonin may also confer indirect renoprotection by modulating circadian organization, although direct evidence that melatonin restores renal-clock-gene amplitude and phase in uremic kidney tissue remains limited [9,17,92]. CKD and end-stage kidney disease are associated with circadian sleep–wake disruption and impaired endogenous melatonin rhythmicity, supporting the biological plausibility that melatonin replacement may act as a chronobiotic intervention in this population [9,92]. In experimental circadian systems, melatonin has been shown to reset clock timing through melatonin-receptor-dependent signaling and E-box-mediated transcriptional regulation of Per1 and Per2, providing a mechanistic basis for its chronobiotic effects [93,94]. Renal circadian clocks regulate kidney physiology, including rhythmic water and electrolyte handling, sodium transport, and expression of renal transport systems; therefore, restoration of CLOCK:BMAL1 rhythmicity could plausibly influence sodium balance and solute clearance [17,95,96]. Circadian clock genes directly regulate renal NHE3 expression, while nephron-specific Bmal1 disruption alters renal organic anion transporter activity, including OAT3, suggesting that clock restoration may affect tubular sodium handling and organic anion/uremic solute transport [95,96,97]. Melatonin’s chronobiotic properties distinguish it from purely symptomatic treatments and support its potential role as a mechanistically relevant therapy for restoring circadian rhythm disruption in CKD [98,99,100].

**Figure 4:**
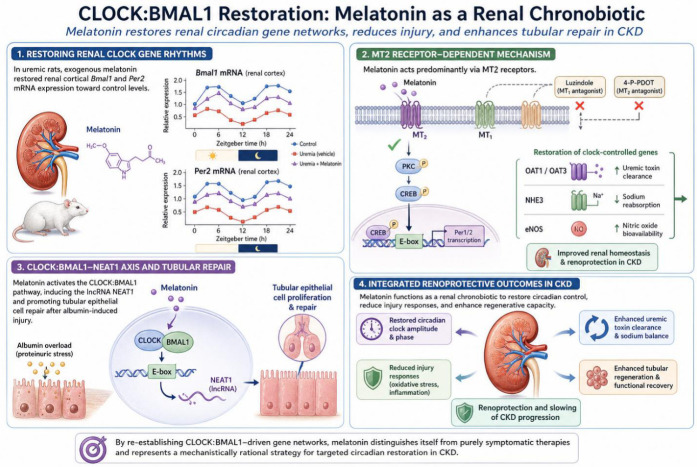
Multimodal renoprotective signaling pathways of melatonin in CKD. This figure summarizes the major molecular pathways through which melatonin may counteract CKD-associated injury. In CKD, uremic toxins and oxidative stress activate inflammatory, inflammasome, fibrotic, mitochondrial, and circadian-disruptive pathways, including NF-κB, NLRP3, TGF-β1/Smad2/3, PI3K/Akt/mTOR dysregulation, impaired SIRT1/PGC-1α/AMPK signaling, and CLOCK:BMAL1 suppression. Melatonin acts through MT1/MT2-dependent and receptor-independent antioxidant mechanisms to enhance Nrf2/HO-1 signaling, suppress NF-κB-mediated inflammation, inhibit NLRP3 inflammasome activation and pyroptosis, reduce TGF-β1/Smad-driven fibrosis, restore autophagy and mitophagy, enhance mitochondrial biogenesis through SIRT1/PGC-1α/AMPK, and potentially support CLOCK:BMAL1-regulated renal repair programs. These integrated actions position melatonin as a candidate antioxidant, anti-inflammatory, anti-fibrotic, mitochondrial-protective, and chronobiotic adjunct in CKD; however, several pathways remain mechanistically provisional and require direct validation in CKD-specific models.

A prior albumin-overload study provides preliminary mechanistic evidence that melatonin can interact with renal-clock-controlled repair pathways, but it should not be interpreted as definitive proof that melatonin broadly restores circadian control of tubular repair programs in CKD. In that model, melatonin activated the clock-controlled long non-coding RNA NEAT1 through a CLOCK:BMAL1-associated pathway and promoted tubular epithelial proliferation after albumin-induced injury [101]. Because this evidence derives from a single research group and from a specific proteinuric tubular-stress model, the CLOCK:BMAL1–NEAT1 axis is best viewed as a hypothesis-generating mechanism requiring replication across independent laboratories, additional CKD models, and time-resolved in vivo renal clock studies. In the broader CKD framework, these findings suggest—but do not prove—that melatonin may support circadian gene networks, reduce injury responses, and enhance regenerative capacity in tubular epithelial cells [59,102] (Figure 4).

## 6. Clinical Evidence for Melatonin in CKD: A Critical Synthesis

### 6.1. Summary of Randomized Controlled Trials

Collectively, randomized controlled trials suggest that melatonin may provide short-term adjunctive benefits in CKD and hemodialysis populations, particularly for sleep disturbance, circadian dysregulation, oxidative stress, inflammation, and selected surrogate endpoints. Most hemodialysis studies used 3 mg nightly for 6 weeks to 3 months, showing improved sleep quality, kidney-disease-specific quality-of-life sleep domains, or circadian rhythm stabilization, with no major safety concerns reported [103,104]. In non-dialysis CKD patients undergoing coronary angiography, Kusirisin et al. used 10 mg twice daily and reported reduced contrast-induced AKI together with improved mitochondrial function and oxidative-stress markers [105]. Marzougui et al. further suggested that combining melatonin with intradialytic exercise may enhance reductions in MDA, TNF-α, and IL-6 compared with exercise alone [91]. However, these studies were small, short in duration, and not powered to assess eGFR slope, proteinuria, kidney failure, cardiovascular events, or mortality. Therefore, melatonin should be regarded as a promising but unproven adjunctive intervention for CKD, rather than as an established renoprotective therapy (Table 2).

Taken together, the available evidence supports melatonin as a low-risk candidate adjunct for sleep, circadian, oxidative, and inflammatory dysfunction in CKD and hemodialysis patients; however, renal function preservation remains unproven, and larger, longer-duration trials with prespecified renal endpoints are required.

### 6.2. Limitations and Gaps in the Current Clinical Evidence

Despite the encouraging mechanistic rationale and generally favorable short-term safety profile, several critical gaps limit translation into practice-changing recommendations. First, no published trial has used renal function trajectory, such as eGFR slope over 12–24 months, or proteinuria as a primary endpoint; available RCTs have largely evaluated surrogate outcomes, including sleep, blood pressure, oxidative-stress markers, and inflammatory biomarkers. Second, dosing has been empirically selected, typically 3–5 mg nightly in hemodialysis studies, rather than pharmacokinetically optimized for CKD, where altered CYP1A2 metabolism and impaired metabolite elimination may affect exposure. Third, no trial has stratified participants by circadian phase, stress burden, endogenous melatonin deficiency, or urinary aMT6s profile. Fourth, chronotherapeutic timing—administration relative to individual circadian phase rather than fixed clock time—has not been tested in CKD populations. From a preventive-medicine perspective, melatonin-containing drugs may eventually be studied as circadian-supportive interventions in high-risk patients with hypertension, diabetes, sleep disturbance, stress-related circadian disruption, or early CKD; however, such use should currently be considered investigational. Finally, several mechanistic proposals in this review—including the OAT1/OAT3–indoxyl sulfate–clock loop, PBMC BMAL1 as a surrogate marker of intrarenal clock status, and combination chronotherapy with SGLT2 inhibitors, finerenone, or RAAS blockade—remain hypotheses that require direct pharmacodynamic, pharmacokinetic, and CKD-specific validation before clinical deployment.

## 7. Chrono-Nephrology: An Emerging Therapeutic Framework

The convergence of evidence reviewed above positions chrono-nephrology—the systematic integration of circadian biology into renal disease research and management—as an emerging conceptual framework rather than an established clinical paradigm. Three translational opportunities merit cautious prioritization.

Urinary aMT6s as a circadian biomarker. Urinary 6-sulfatoxymelatonin is a validated, non-invasive index of nocturnal melatonin secretion. Incorporating aMT6s measurement into CKD staging could identify high-risk patients with severe melatonin deficiency for targeted supplementation trials, analogously to the role of HbA1c in guiding insulin therapy in diabetes.

BMAL1 in peripheral blood mononuclear cells as an exploratory clock-restoration biomarker. Time-of-day sampling of PBMC BMAL1 rhythmicity could be explored as a pharmacodynamic endpoint in future melatonin chronotherapy trials. However, the translational validity of PBMC clock gene expression as a surrogate for intrarenal clock amplitude in humans has not been established. PBMC BMAL1 should therefore be considered a candidate research biomarker requiring validation against renal tissue, urinary markers, and clinical endpoints, not as a ready-to-deploy clinical endpoint.

Combination chronotherapy as a testable hypothesis. Melatonin may theoretically complement SGLT2 inhibitors, finerenone, and evening-dosed RAAS inhibitors through partially distinct effects on AMPK signaling, inflammation, oxidative stress, and nocturnal blood pressure control. However, pharmacokinetic and pharmacodynamic interactions between melatonin and these agents in CKD have not been systematically examined. Accordingly, combination chronotherapy is now framed as a hypothesis that warrants stepwise preclinical testing and safety evaluation before clinical trial design.

## 8. Conclusions

In conclusion, circadian rhythm disruption and melatonin deficiency appear to be important components of CKD pathophysiology, driven by uremic, inflammatory, oxidative, and metabolic signaling that suppresses renal clock function and may perpetuate disease progression. Melatonin may counteract selected components of these processes through antioxidant, anti-inflammatory, anti-fibrotic, mitochondrial, and circadian mechanisms, but several mechanistic links remain provisional and require direct CKD-specific validation. Current clinical evidence supports short-term safety and potential efficacy of nightly melatonin supplementation for sleep quality and selected oxidative or inflammatory surrogate endpoints in dialysis patients, particularly when combined with exercise. Nevertheless, whether melatonin can slow CKD progression, reduce proteinuria, preserve eGFR, or improve hard renal and cardiovascular outcomes remains uncertain. Future studies should include adequately powered long-term designs, time-resolved circadian biomarker sampling, pharmacokinetic assessment in CKD, and clinically meaningful renal endpoints. Collectively, these findings support chrono-nephrology as a promising research framework and melatonin as a candidate adjunctive strategy rather than an established disease-modifying therapy.

## Figures and Tables

**Table 1:** Principal modes of melatonin action in renal tissue, organized by receptor-dependent and receptor-independent pathways.

Receptor/Mode	Signaling Pathway	Key Renal Action	References
MT1 (Gαi-coupled GPCR)	Gi/o → ↓ cAMP/PKA; Gβγ → PLC/IP_3_/Ca^2+^; ↑ ERK1/2; ↑ PI3K/Akt	Afferent arteriolar vasoconstriction; tubular Ca^2+^ reabsorption; SCN circadian phase shifting	[37,38,39]
MT2 (Gαi-coupled GPCR)	Gi/o → ↓ cAMP and ↓ cGMP; ↑ PKC; ↑ STAT6	M2 macrophage polarization; anti-inflammatory; renin suppression in juxtaglomerular cells	[38,40]
MT3/NQO2	Direct transactivation of *ARE/RORE* genes	Nrf2, Bmal1, HSD3B6 transcription; antioxidant gene activation	[41]
Receptor-independent	Direct scavenging of •OH and ONOO^−^ → AFMK → AMK cascade	Non-enzymatic antioxidant action; particularly relevant within the mitochondrial matrix (~10× plasma concentration)	[42,43,44,45]

**Table 2:** Selected randomized controlled trials of melatonin in CKD and hemodialysis populations.

Study (Year)	Population and Dose	Key Outcomes
Kusirisin et al. (2025) [105]	CKD patients (eGFR < 60 mL/min/1.73 m^2^) undergoing coronary angiography (n = 40); 10 mg twice daily	Reduced incidence of contrast-induced AKI (25% vs. 60%; *p* = 0.025); enhanced mitochondrial function and reduced oxidative stress
Larki et al. (2025) [103]	Hemodialysis patients (n = 82); 3 mg nightly × 6 weeks	Significant improvement in sleep quality (PSQI) and the kidney-disease-specific QoL sleep domain (*p* = 0.003); no significant effect on BP
Marzougui et al. (2024) [91]	Hemodialysis patients (n = 32 across 3 arms); 3 mg nightly + intradialytic concurrent training × 12 weeks	Synergistic reductions in MDA, TNF-α, and IL-6; significantly greater oxidative-stress reduction with combined therapy than with exercise alone
Russcher et al., MELODY (2013) [104]	Hemodialysis patients (n = 30); 3 mg nightly × 3 months	Long-term maintenance of QoL and sleep; no significant adverse events at 3 mg; established safety precedent
Koch et al., EMSCAP (2009) [106]	Hemodialysis patients (crossover design); 3 mg nightly	Normalization of circadian rhythm; improved sleep quality; reference standard for hemodialysis melatonin dosing

## Data Availability

No new datasets were generated or analyzed during the preparation of this review; all supporting data are derived from the published studies cited herein.

## Abbreviations

AANAT, arylalkylamine N-acetyltransferase; AKI, acute kidney injury; AMPK, AMP-activated protein kinase; ARE, antioxidant response element; BMAL1, brain and muscle ARNT-like protein 1; CCG, clock-controlled gene; CKD, chronic kidney disease; CKD-MBD, CKD–mineral and bone disorder; CK1δ/ε, casein kinase 1 delta/epsilon; CLOCK, circadian locomotor output cycles kaput; CRY, cryptochrome; eNOS, endothelial nitric oxide synthase; ESRD, end-stage renal disease; GFR, glomerular filtration rate; GSDMD-N, gasdermin D N-terminal fragment; HA, hippuric acid; HO-1, heme oxygenase-1; IS, indoxyl sulfate; MT1/MT2, melatonin receptor type 1/2; mTOR, mammalian target of rapamycin; NF-κB, nuclear factor kappa-light-chain-enhancer of activated B cells; NLRP3, NOD-, LRR-, and pyrin domain-containing protein 3; NQO1, NAD(P)H quinone dehydrogenase 1; Nrf2, nuclear factor erythroid 2-related factor 2; PER, Period; pCS, p-cresyl sulfate; PSQI, Pittsburgh Sleep Quality Index; RAAS, renin–angiotensin–aldosterone system; REV-ERB, reverse strand of erb-B2; RORα, RAR-related orphan receptor alpha; ROS, reactive oxygen species; SCN, suprachiasmatic nucleus; SIRT1, sirtuin 1; Smad, small mothers against decapentaplegic; TGF-β1, transforming growth factor beta 1; TTFL, transcription–translation feedback loop; UUO, unilateral ureteral obstruction; δΨm, mitochondrial membrane potential.

## References

[1] GBD Chronic Kidney Disease Collaboration (2020). Global, regional, and national burden of chronic kidney disease, 1990–2017: A systematic analysis for the Global Burden of Disease Study 2017. Lancet.

[2] Contreras G., Pagan J., Elfassy T., Bandes M., Bustamante N., Ortigosa-Goggins M., Becerra L.A., Munoz Mendoza J., Drexler Y., Alonso S. (2025). Trends of preemptive kidney transplantation in lupus compared to other primary diseases leading to end stage kidney disease in the United States of America. Lupus.

[3] Firsov D., Bonny O. (2018). Circadian rhythms and the kidney. Nat. Rev. Nephrol..

[4] Gumz M.L., Stow L.R., Lynch I.J., Greenlee M.M., Rudin A., Cain B.D., Weaver D.R., Wingo C.S. (2009). The circadian clock protein Period 1 regulates expression of the renal epithelial sodium channel in mice. J. Clin. Investig..

[5] Jamadar A., Ward C.J., Remadevi V., Varghese M.M., Pabla N.S., Gumz M.L., Rao R. (2025). Circadian Clock Disruption and Growth of Kidney Cysts in Autosomal Dominant Polycystic Kidney Disease. J. Am. Soc. Nephrol..

[6] Egstrand S., Mace M.L., Olgaard K., Lewin E. (2021). The Vascular Circadian Clock in Chronic Kidney Disease. Cells.

[7] Ohashi N., Ishigaki S., Isobe S., Matsuyama T., Sato T., Fujikura T., Tsuji T., Kato A., Yasuda H. (2019). Salt Loading Aggravates the Relationship between Melatonin and Proteinuria in Patients with Chronic Kidney Disease. Intern. Med..

[8] Ludemann P., Zwernemann S., Lerchl A. (2001). Clearance of melatonin and 6-sulfatoxymelatonin by hemodialysis in patients with end-stage renal disease. J. Pineal Res..

[9] Koch B.C., Nagtegaal J.E., Kerkhof G.A., ter Wee P.M. (2009). Circadian sleep-wake rhythm disturbances in end-stage renal disease. Nat. Rev. Nephrol..

[10] Juffre A., Gumz M.L. (2024). Recent advances in understanding the kidney circadian clock mechanism. Am. J. Physiol.-Ren. Physiol..

[11] Reiter R.J., Tan D.X., Rosales-Corral S., Manchester L.C. (2013). The universal nature, unequal distribution and antioxidant functions of melatonin and its derivatives. Mini Rev. Med. Chem..

[12] Tomatsu S., Abbott S.M., Attarian H. (2025). Clinical Chronobiology: Circadian Rhythms in Health and Disease. Semin. Neurol..

[13] Van Drunen R., Eckel-Mahan K. (2021). Circadian Rhythms of the Hypothalamus: From Function to Physiology. Clocks Sleep.

[14] Zha K., Mi B., Xiong Y., Wu S., Lu L., Zhang S., Lu X., Mak H.C., Huang J., Panayi A.C. (2025). Circadian Rhythm: Biological Functions, Diseases, and Therapeutic Targets. MedComm.

[15] Healy K.L., Morris A.R., Liu A.C. (2021). Circadian Synchrony: Sleep, Nutrition, and Physical Activity. Front. Netw. Physiol..

[16] Takahashi J.S. (2017). Transcriptional architecture of the mammalian circadian clock. Nat. Rev. Genet..

[17] Costello H.M., Johnston J.G., Juffre A., Crislip G.R., Gumz M.L. (2022). Circadian clocks of the kidney: Function, mechanism, and regulation. Physiol. Rev..

[18] Mohandas R., Douma L.G., Scindia Y., Gumz M.L. (2022). Circadian rhythms and renal pathophysiology. J. Clin. Investig..

[19] Nikolaeva S., Ansermet C., Centeno G., Pradervand S., Bize V., Mordasini D., Henry H., Koesters R., Maillard M., Bonny O. (2016). Nephron-Specific Deletion of Circadian Clock Gene Bmal1 Alters the Plasma and Renal Metabolome and Impairs Drug Disposition. J. Am. Soc. Nephrol..

[20] Saifur Rohman M., Emoto N., Nonaka H., Okura R., Nishimura M., Yagita K., van der Horst G.T., Matsuo M., Okamura H., Yokoyama M. (2005). Circadian clock genes directly regulate expression of the Na(+)/H(+) exchanger NHE3 in the kidney. Kidney Int..

[21] Richards J., Ko B., All S., Cheng K.Y., Hoover R.S., Gumz M.L. (2014). A role for the circadian clock protein Per1 in the regulation of the NaCl co-transporter (NCC) and the with-no-lysine kinase (WNK) cascade in mouse distal convoluted tubule cells. J. Biol. Chem..

[22] Zhang D., Jin C., Obi I.E., Rhoads M.K., Soliman R.H., Sedaka R.S., Allan J.M., Tao B., Speed J.S., Pollock J.S. (2020). Loss of circadian gene Bmal1 in the collecting duct lowers blood pressure in male, but not female, mice. Am. J. Physiol.-Ren. Physiol..

[23] Richards J., Gumz M.L. (2012). Advances in understanding the peripheral circadian clocks. FASEB J..

[24] Carriazo S., Ramos A.M., Sanz A.B., Sanchez-Nino M.D., Kanbay M., Ortiz A. (2020). Chronodisruption: A Poorly Recognized Feature of CKD. Toxins.

[25] Ohashi N., Isobe S., Ishigaki S., Yasuda H. (2017). Circadian rhythm of blood pressure and the renin-angiotensin system in the kidney. Hypertens. Res..

[26] Jeong J.H., Fonkoue I.T., Quyyumi A.A., DaCosta D., Park J. (2020). Nocturnal blood pressure is associated with sympathetic nerve activity in patients with chronic kidney disease. Physiol. Rep..

[27] Borrelli S., Garofalo C., Gabbai F.B., Chiodini P., Signoriello S., Paoletti E., Ravera M., Bussalino E., Bellizzi V., Liberti M.E. (2023). Dipping Status, Ambulatory Blood Pressure Control, Cardiovascular Disease, and Kidney Disease Progression: A Multicenter Cohort Study of CKD. Am. J. Kidney Dis..

[28] Rapa S.F., Di Iorio B.R., Campiglia P., Heidland A., Marzocco S. (2019). Inflammation and Oxidative Stress in Chronic Kidney Disease-Potential Therapeutic Role of Minerals, Vitamins and Plant-Derived Metabolites. Int. J. Mol. Sci..

[29] Chen X., Zhang W., Gu Y., Huang S. (2025). Circadian clocks and their role in kidney and eye diseases across organ systems. Front. Physiol..

[30] Hardeland R. (2010). Melatonin metabolism in the central nervous system. Curr. Neuropharmacol..

[31] Hardeland R. (2022). Redox Biology of Melatonin: Discriminating Between Circadian and Noncircadian Functions. Antioxid. Redox Signal..

[32] Sevilla A., Cheret J., Slominski R.M., Slominski A.T., Paus R. (2022). Revisiting the role of melatonin in human melanocyte physiology: A skin context perspective. J. Pineal Res..

[33] Gu P., Wu Y., Lu W. (2024). New Perspectives on the Role and Therapeutic Potential of Melatonin in Cardiovascular Diseases. Am. J. Cardiovasc. Drugs.

[34] Su W.L., Wu C.C., Wu S.V., Lee M.C., Liao M.T., Lu K.C., Lu C.L. (2022). A Review of the Potential Effects of Melatonin in Compromised Mitochondrial Redox Activities in Elderly Patients With COVID-19. Front. Nutr..

[35] Inigo-Catalina L., Ortiz-Cabello M., Navarro E., Esteras N., Rancan L., Paredes S.D. (2025). Melatonin-Mediated Nrf2 Activation as a Potential Therapeutic Strategy in Mutation-Driven Neurodegenerative Diseases. Antioxidants.

[36] Lavado-Fernandez E., Perez-Montes C., Robles-Garcia M., Santos-Ledo A., Garcia-Macia M. (2026). Melatonin at the Crossroads of Oxidative Stress, Immunity, and Cancer Therapy. Antioxidants.

[37] Dubocovich M.L., Delagrange P., Krause D.N., Sugden D., Cardinali D.P., Olcese J. (2010). International Union of Basic and Clinical Pharmacology. LXXV. Nomenclature, classification, and pharmacology of G protein-coupled melatonin receptors. Pharmacol. Rev..

[38] Liu J., Clough S.J., Hutchinson A.J., Adamah-Biassi E.B., Popovska-Gorevski M., Dubocovich M.L. (2016). MT1 and MT2 Melatonin Receptors: A Therapeutic Perspective. Annu. Rev. Pharmacol. Toxicol..

[39] Dubocovich M.L., Hudson R.L., Sumaya I.C., Masana M.I., Manna E. (2005). Effect of MT1 melatonin receptor deletion on melatonin-mediated phase shift of circadian rhythms in the C57BL/6 mouse. J. Pineal Res..

[40] Yi W.J., Kim T.S. (2017). Melatonin protects mice against stress-induced inflammation through enhancement of M2 macrophage polarization. Int. Immunopharmacol..

[41] Nosjean O., Ferro M., Coge F., Beauverger P., Henlin J.M., Lefoulon F., Fauchere J.L., Delagrange P., Canet E., Boutin J.A. (2000). Identification of the melatonin-binding site MT3 as the quinone reductase 2. J. Biol. Chem..

[42] Reiter R.J., Rosales-Corral S., Tan D.X., Jou M.J., Galano A., Xu B. (2017). Melatonin as a mitochondria-targeted antioxidant: One of evolution’s best ideas. Cell. Mol. Life Sci..

[43] Galano A., Tan D.X., Reiter R.J. (2013). On the free radical scavenging activities of melatonin’s metabolites, AFMK and AMK. J. Pineal Res..

[44] Tan D.X., Manchester L.C., Qin L., Reiter R.J. (2016). Melatonin: A Mitochondrial Targeting Molecule Involving Mitochondrial Protection and Dynamics. Int. J. Mol. Sci..

[45] Reiter R.J., Mayo J.C., Tan D.X., Sainz R.M., Alatorre-Jimenez M., Qin L. (2016). Melatonin as an antioxidant: Under promises but over delivers. J. Pineal Res..

[46] Xiong Y., Zhong Q., Zhang Y., Liu Z., Wang X. (2024). The association between circadian syndrome and chronic kidney disease in an aging population: A 4-year follow-up study. Front. Endocrinol..

[47] Fang Y., Jo S.K., Park S.J., Yang J., Ko Y.S., Lee H.Y., Oh S.W., Cho W.Y., Kim K., Son G.H. (2023). Role of the Circadian Clock and Effect of Time-Restricted Feeding in Adenine-Induced Chronic Kidney Disease. Lab. Investig..

[48] Xie H., Yang N., Yu C., Lu L. (2024). Uremic toxins mediate kidney diseases: The role of aryl hydrocarbon receptor. Cell. Mol. Biol. Lett..

[49] Yang S., Ye Z., Chen L., Zhou X., Li W., Cheng F. (2025). Circadian Clock Gene Bmal1: A Molecular Bridge from AKI to CKD. Biomolecules.

[50] Wu W., Bush K.T., Nigam S.K. (2017). Key Role for the Organic Anion Transporters, OAT1 and OAT3, in the in vivo Handling of Uremic Toxins and Solutes. Sci. Rep..

[51] Li Q., Holzwarth J.A., Smith B., Karaz S., Membrez M., Sorrentino V., Summers S., Spears J., Migliavacca E. (2024). Impaired renal transporter gene expression and uremic toxin excretion as aging hallmarks in cats with naturally occurring chronic kidney disease. Aging.

[52] Aranda-Rivera A.K., Cruz-Gregorio A., Pedraza-Chaverri J., Scholze A. (2022). Nrf2 Activation in Chronic Kidney Disease: Promises and Pitfalls. Antioxidants.

[53] Saito H. (2013). Toxico-pharmacological perspective of the Nrf2-Keap1 defense system against oxidative stress in kidney diseases. Biochem. Pharmacol..

[54] Ebert T., Neytchev O., Witasp A., Kublickiene K., Stenvinkel P., Shiels P.G. (2021). Inflammation and Oxidative Stress in Chronic Kidney Disease and Dialysis Patients. Antioxid. Redox Signal..

[55] Zhou H., Li D., Zhu P., Hu S., Hu N., Ma S., Zhang Y., Han T., Ren J., Cao F. (2017). Melatonin suppresses platelet activation and function against cardiac ischemia/reperfusion injury via PPARgamma/FUNDC1/mitophagy pathways. J. Pineal Res..

[56] El Agaty S.M., Khedr S., Mostafa D.K.M., Wanis N.A., Abou-Bakr D.A. (2024). Protective role of melatonin against diclofenac-induced acute kidney injury. Life Sci..

[57] Bonomini F., Dos Santos M., Veronese F.V., Rezzani R. (2019). NLRP3 Inflammasome Modulation by Melatonin Supplementation in Chronic Pristane-Induced Lupus Nephritis. Int. J. Mol. Sci..

[58] Liu H., Xiang X., Shi C., Guo J., Ran T., Lin J., Dong F., Yang J., Miao H. (2025). Oxidative stress and inflammation in renal fibrosis: Novel molecular mechanisms and therapeutic targets. Chem. Biol. Interact..

[59] Xu Y.Y., Chen T., Ding H., Chen Q., Fan Q.L. (2024). Melatonin inhibits circadian gene DEC1 and TLR2/MyD88/NF-kappaB signaling pathway to alleviate renal injury in type 2 diabetic mice. Acta Diabetol..

[60] Fan Z., Qi X., Yang W., Xia L., Wu Y. (2020). Melatonin Ameliorates Renal Fibrosis Through the Inhibition of NF-kappaB and TGF-beta1/Smad3 Pathways in db/db Diabetic Mice. Arch. Med. Res..

[61] Lin Y.W., Lee L.M., Lee W.J., Chu C.Y., Tan P., Yang Y.C., Chen W.Y., Yang S.F., Hsiao M., Chien M.H. (2016). Melatonin inhibits MMP-9 transactivation and renal cell carcinoma metastasis by suppressing Akt-MAPKs pathway and NF-kappaB DNA-binding activity. J. Pineal Res..

[62] Swanson K.V., Deng M., Ting J.P. (2019). The NLRP3 inflammasome: Molecular activation and regulation to therapeutics. Nat. Rev. Immunol..

[63] Rivera R.F., Sciarrone Alibrandi M.T., Foligno N.E., Magagnoli L., Ciceri P., Cozzolino M. (2026). Uremic Toxin-Driven Vascular Calcification in Chronic Kidney Disease: Molecular Pathways and Integrated Phenotypes. Toxins.

[64] Gifford C.C., Tang J., Costello A., Khakoo N.S., Nguyen T.Q., Goldschmeding R., Higgins P.J., Samarakoon R. (2021). Negative regulators of TGF-beta1 signaling in renal fibrosis; pathological mechanisms and novel therapeutic opportunities. Clin. Sci..

[65] Lan H.Y., Chung A.C. (2012). TGF-beta/Smad signaling in kidney disease. Semin. Nephrol..

[66] Hosseinzadeh A., Pourhanifeh M.H., Amiri S., Sheibani M., Irilouzadian R., Reiter R.J., Mehrzadi S. (2024). Therapeutic potential of melatonin in targeting molecular pathways of organ fibrosis. Pharmacol. Rep..

[67] Hu W., Ma Z., Jiang S., Fan C., Deng C., Yan X., Di S., Lv J., Reiter R.J., Yang Y. (2016). Melatonin: The dawning of a treatment for fibrosis?. J. Pineal Res..

[68] Ma Z., Xin Z., Di W., Yan X., Li X., Reiter R.J., Yang Y. (2017). Melatonin and mitochondrial function during ischemia/reperfusion injury. Cell. Mol. Life Sci..

[69] Chen D.Q., Cao G., Zhao H., Chen L., Yang T., Wang M., Vaziri N.D., Guo Y., Zhao Y.Y. (2019). Combined melatonin and poricoic acid A inhibits renal fibrosis through modulating the interaction of Smad3 and beta-catenin pathway in AKI-to-CKD continuum. Ther. Adv. Chronic Dis..

[70] Huang Y.S., Lu K.C., Chao T.K., Chen J.S., Chen A., Guo C.Y., Hsieh H.Y., Shih H.M., Sytwu H.K., Wu C.C. (2018). Role of melatonin receptor 1A and pituitary homeobox-1 coexpression in protecting tubular epithelial cells in membranous nephropathy. J. Pineal Res..

[71] Kim J.Y., Park J.H., Jeon E.J., Leem J., Park K.K. (2020). Melatonin Prevents Transforming Growth Factor-beta1-Stimulated Transdifferentiation of Renal Interstitial Fibroblasts to Myofibroblasts by Suppressing Reactive Oxygen Species-Dependent Mechanisms. Antioxidants.

[72] Han Y.S., Yoon Y.M., Go G., Lee J.H., Lee S.H. (2020). Melatonin Protects Human Renal Proximal Tubule Epithelial Cells Against High Glucose-Mediated Fibrosis via the Cellular Prion Protein-TGF-beta-Smad Signaling Axis. Int. J. Med. Sci..

[73] Adelibieke Y., Shimizu H., Saito S., Mironova R., Niwa T. (2013). Indoxyl sulfate counteracts endothelial effects of erythropoietin through suppression of Akt phosphorylation. Circ. J..

[74] Ito S., Osaka M., Edamatsu T., Itoh Y., Yoshida M. (2016). Crucial Role of the Aryl Hydrocarbon Receptor (AhR) in Indoxyl Sulfate-Induced Vascular Inflammation. J. Atheroscler. Thromb..

[75] Godel M., Hartleben B., Herbach N., Liu S., Zschiedrich S., Lu S., Debreczeni-Mor A., Lindenmeyer M.T., Rastaldi M.P., Hartleben G. (2011). Role of mTOR in podocyte function and diabetic nephropathy in humans and mice. J. Clin. Investig..

[76] Liu L., Yang L., Chang B., Zhang J., Guo Y., Yang X. (2018). The protective effects of rapamycin on cell autophagy in the renal tissues of rats with diabetic nephropathy via mTOR-S6K1-LC3II signaling pathway. Ren. Fail..

[77] Chen W.R., Yang J.Q., Liu F., Shen X.Q., Zhou Y.J. (2020). Melatonin attenuates vascular calcification by activating autophagy via an AMPK/mTOR/ULK1 signaling pathway. Exp. Cell Res..

[78] Tang H., Yang M., Liu Y., Zhu X., Liu S., Liu H., Sun L., Song P. (2022). Melatonin alleviates renal injury by activating mitophagy in diabetic nephropathy. Front. Endocrinol..

[79] Luo N., Wang Y., Ma Y., Liu Y., Liu Z. (2023). Melatonin alleviates renal injury in diabetic rats by regulating autophagy. Mol. Med. Rep..

[80] Ozbek E., Ilbey Y.O., Ozbek M., Simsek A., Cekmen M., Somay A. (2009). Melatonin attenuates unilateral ureteral obstruction-induced renal injury by reducing oxidative stress, iNOS, MAPK, and NF-kB expression. J. Endourol..

[81] Liu X., Luo D., Huang S., Liu S., Zhang B., Wang F., Lu J., Chen J., Li S. (2021). Impaired Nicotinamide Adenine Dinucleotide Biosynthesis in the Kidney of Chronic Kidney Disease. Front. Physiol..

[82] Morevati M., Egstrand S., Nordholm A., Mace M.L., Andersen C.B., Salmani R., Olgaard K., Lewin E. (2021). Effect of NAD+ boosting on kidney ischemia-reperfusion injury. PLoS ONE.

[83] Guo P., Pi H., Xu S., Zhang L., Li Y., Li M., Cao Z., Tian L., Xie J., Li R. (2014). Melatonin Improves mitochondrial function by promoting MT1/SIRT1/PGC-1 alpha-dependent mitochondrial biogenesis in cadmium-induced hepatotoxicity in vitro. Toxicol. Sci..

[84] Qi X., Wang J. (2020). Melatonin improves mitochondrial biogenesis through the AMPK/PGC1alpha pathway to attenuate ischemia/reperfusion-induced myocardial damage. Aging.

[85] Canto C., Gerhart-Hines Z., Feige J.N., Lagouge M., Noriega L., Milne J.C., Elliott P.J., Puigserver P., Auwerx J. (2009). AMPK regulates energy expenditure by modulating NAD+ metabolism and SIRT1 activity. Nature.

[86] Asher G., Gatfield D., Stratmann M., Reinke H., Dibner C., Kreppel F., Mostoslavsky R., Alt F.W., Schibler U. (2008). SIRT1 regulates circadian clock gene expression through PER2 deacetylation. Cell.

[87] Nakahata Y., Kaluzova M., Grimaldi B., Sahar S., Hirayama J., Chen D., Guarente L.P., Sassone-Corsi P. (2008). The NAD+-dependent deacetylase SIRT1 modulates CLOCK-mediated chromatin remodeling and circadian control. Cell.

[88] Peek C.B., Affinati A.H., Ramsey K.M., Kuo H.Y., Yu W., Sena L.A., Ilkayeva O., Marcheva B., Kobayashi Y., Omura C. (2013). Circadian clock NAD+ cycle drives mitochondrial oxidative metabolism in mice. Science.

[89] Marzougui H., Hammouda O., Ben Dhia I., Maaloul R., Agrebi I., Chaker H., Kammoun K., Ben Hmida M., Ayadi F., Kallel C. (2021). Melatonin ingestion before intradialytic exercise improves immune responses in hemodialysis patients. Int. Urol. Nephrol..

[90] Marzougui H., Turki M., Ben Dhia I., Maaloul R., Chaker H., Makhlouf R., Agrebi I., Kammoun K., Jamoussi K., Ayadi F. (2023). Melatonin intake before intradialytic exercise reverses oxidative stress and improves antioxidant status in hemodialysis patients. Int. J. Artif. Organs.

[91] Marzougui H., Ben Dhia I., Mezghani I., Maaloul R., Toumi S., Kammoun K., Chaabouni M.N., Ayadi F., Ben Hmida M., Turki M. (2024). The Synergistic Effect of Intradialytic Concurrent Training and Melatonin Supplementation on Oxidative Stress and Inflammation in Hemodialysis Patients: A Double-Blind Randomized Controlled Trial. Antioxidants.

[92] Koch B.C., van der Putten K., Van Someren E.J., Wielders J.P., Ter Wee P.M., Nagtegaal J.E., Gaillard C.A. (2010). Impairment of endogenous melatonin rhythm is related to the degree of chronic kidney disease (CREAM study). Nephrol. Dial. Transplant..

[93] Kandalepas P.C., Mitchell J.W., Gillette M.U. (2016). Melatonin Signal Transduction Pathways Require E-Box-Mediated Transcription of Per1 and Per2 to Reset the SCN Clock at Dusk. PLoS ONE.

[94] Hunt A.E., Al-Ghoul W.M., Gillette M.U., Dubocovich M.L. (2001). Activation of MT(2) melatonin receptors in rat suprachiasmatic nucleus phase advances the circadian clock. Am. J. Physiol. Cell Physiol..

[95] Zuber A.M., Centeno G., Pradervand S., Nikolaeva S., Maquelin L., Cardinaux L., Bonny O., Firsov D. (2009). Molecular clock is involved in predictive circadian adjustment of renal function. Proc. Natl. Acad. Sci. USA.

[96] Solocinski K., Gumz M.L. (2015). The Circadian Clock in the Regulation of Renal Rhythms. J. Biol. Rhythm..

[97] Dutta P., Layton A.T. (2024). Sex and circadian regulation of metabolic demands in the rat kidney: A modeling analysis. PLoS ONE.

[98] Rahman A., Hasan A.U., Kobori H. (2019). Melatonin in chronic kidney disease: A promising chronotherapy targeting the intrarenal renin-angiotensin system. Hypertens. Res..

[99] Nagasawa Y., Hasuike Y., Kuragano T., Ishihara M. (2019). Circadian Rhythm and CKD: Is Melatonin a Key Player or Bi-player?. Intern. Med..

[100] Markowska M., Niemczyk S., Romejko K. (2023). Melatonin Treatment in Kidney Diseases. Cells.

[101] Huang Y.S., Lu K.C., Chang Y.T., Ka S.M., Guo C.Y., Hsieh H.Y., Shih H.M., Sytwu H.K., Wu C.C. (2024). Melatonin Alleviates Albumin-Induced Tubular Cell Injury by Activating Clock-Controlled Nuclear Enriched Abundant Transcript 1-Mediated Proliferation. ACS Pharmacol. Transl. Sci..

[102] Chen J., Zhang S., Xue X., Ma X., Chen A., Wu Y., Wang G., Zhang Q., Xue Y., Jia Y. (2025). Melatonin attenuates kidney injury by alleviating lysosomal damage in diabetic kidney disease. Acta Biochim. Biophys. Sin..

[103] Larki R.A., Iranmanesh A., Gholami D., Manzouri L. (2025). The effect of oral melatonin on the quality of life, sleep and blood pressure of hemodialysis patients: A randomized clinical trial. BMC Nephrol..

[104] Russcher M., Koch B.C., Nagtegaal J.E., van Ittersum F.J., Pasker-de Jong P.C., Hagen E.C., van Dorp W.T., Gabreels B., Wildbergh T.X., van der Westerlaken M.M. (2013). Long-term effects of melatonin on quality of life and sleep in haemodialysis patients (Melody study): A randomized controlled trial. Br. J. Clin. Pharmacol..

[105] Kusirisin P., Apaijai N., Noppakun K., Kuanprasert S., Chattipakorn S.C., Chattipakorn N. (2025). Protective Effects of Melatonin on Kidney Function Against Contrast Media-Induced Kidney Damage in Patients With Chronic Kidney Disease: A Prospective, Randomized, Double-Blinded, Placebo-Controlled Trial. J. Pineal Res..

[106] Koch B.C., Nagtegaal J.E., Hagen E.C., van der Westerlaken M.M., Boringa J.B., Kerkhof G.A., Ter Wee P.M. (2009). The effects of melatonin on sleep-wake rhythm of daytime haemodialysis patients: A randomized, placebo-controlled, cross-over study (EMSCAP study). Br. J. Clin. Pharmacol..

